# A rare incidental finding during routine pathological evaluation of gallbladder specimen: a case report

**DOI:** 10.1016/j.ijscr.2025.111367

**Published:** 2025-04-25

**Authors:** Sarra Ben Rejeb, Yasmine Chaabane, Moez Sahnoun, Adnen Chouchen

**Affiliations:** a Pathology Department, Security Forces Hospital, Marsa, Tunisia; b Surgery Department, Security Forces Hospital, Marsa, Tunisia

**Keywords:** Neuroendocrine tumor, Gallbladder, Pathology, Surgery, Cystic duct

## Abstract

**Introduction and importance:**

Neuroendocrine tumors (NETs) of the gallbladder are rare, often discovered incidentally, with unclear pathogenesis and management strategies.

**Case presentation:**

A 51-year-old man with hypertension presented with abdominal pain and a positive Murphy’s sign. After laparoscopic cholecystectomy for suspected acute cholecystitis, histopathology revealed a grade 1 NET at the cystic duct margin, invading the subserosa (pT2). Staging showed no metastases, though pericholedochal lymph nodes were enlarged. A second surgery resected the cystic duct and regional lymph nodes, revealing no residual tumor. No adjuvant therapy was given, and the patient remains disease-free at 5 years.

**Clinical discussion:**

Gallbladder NETs often mimic common biliary conditions, making the preoperative diagnosis challenging due to nonspecific findings. This case highlights the pathology’s key role in the diagnosis of asymptomatic early-stage tumors and suggests a favorable prognosis for well-differentiated NETs with surgical management, despite unclear adjuvant therapy guidelines.

**Conclusion:**

NETs of the gallbladder are uncommon with nonspecific clinical and imaging features. This rare case emphasizes the importance of routine pathological examination of gallbladder specimens in detecting and grading of asymptomatic and early stage tumors such as NETs.

## Highlights

• Rare NET of gallbladder found at cystic duct margin (<0.5% of NETs) during routine histopathology post-cholecystectomy.
• Histopathology and immunohistochemistry key to distinguish NETs from common gallbladder issues despite nonspecific findings.
• Positive margin led to resection of cystic duct and nodes; disease-free at 2 years without adjuvant therapy for low-grade NET.

## 1 Introduction

Neuroendocrine tumors (NETs) of the gallbladder and biliary tract are exceptionally rare, accounting for 0.2 % to 0.5 % of all NETs [[1], [2], [3]]. Their pathogenesis remains poorly understood, with possible links to Von Hippel-Lindau syndrome and multiple endocrine neoplasia type 1 (MEN1) [1,4,5]. Often incidentally discovered during cholecystectomy for benign conditions such as cholelithiasis or cholecystitis,the diagnosis and treatment of NETs of the gallbladder are challenging due to their rarity and nonspecific symptoms. Currently, no specific guidelines exist for these tumors, unlike gastrointestinal NETs, leaving management reliant on case-specific decisions [2,6]. This case, managed at the Security Forces Hospital, a tertiary care center in Marsa, Tunisia, illustrated the critical role of routine histopathology in identifying such tumors.

## 2 Case report

**Fig. 1:**
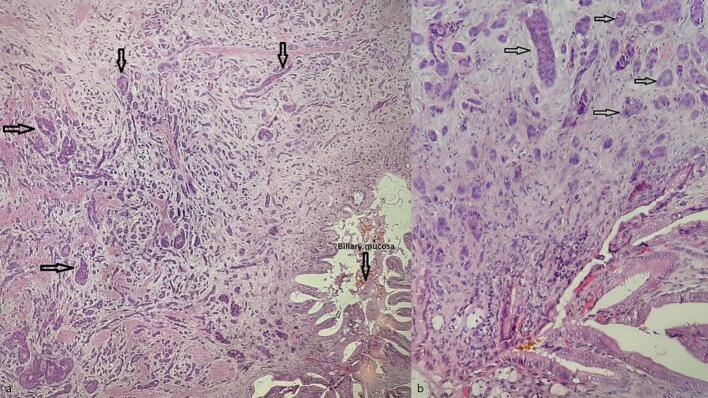
(a) Hematoxylin-eosin staining ×200: Tumor cell proliferation arranged in nests and cords of monotonous cells in the cystic duct wall (b) Hematoxylin-eosin staining ×400: Tumor cells are round-to-oval showing features of neuroendocrine differentiation.

**Fig. 2:**
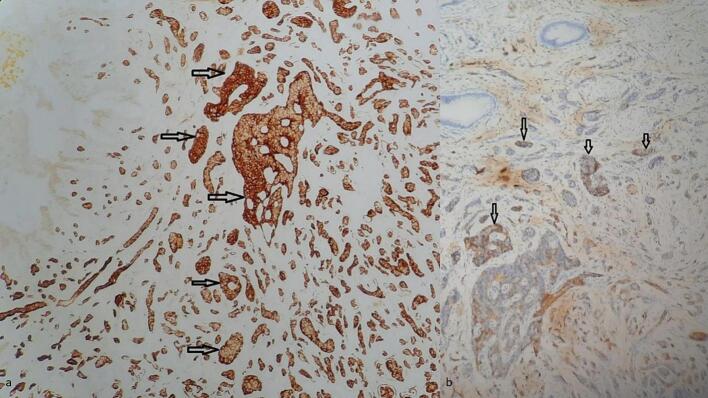
(a) Immunohistochemistry ×100: tumor cells show strong and diffuse staining for chromogranin antibody (b) Immunohistochemistry w 200: tumor cells show positive staining for CD56 antibody.

**Fig. 3:**
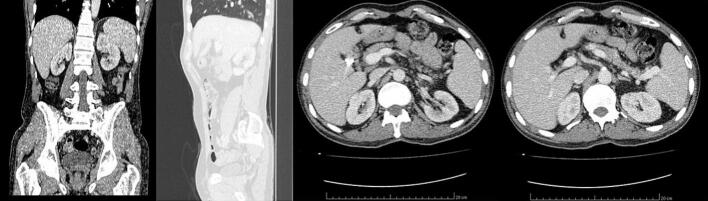
Different coronal slices of the post-operative abdominal CT scans showing no recurrence or metastases.

**Fig. 4:**
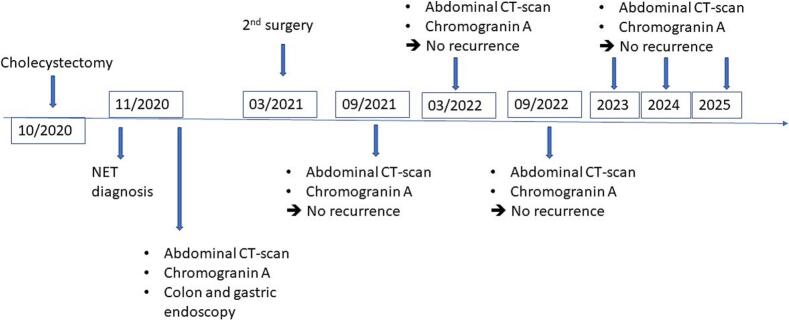
Patient timeline. Patient timeline showing initial presentation in October 2020, NET diagnosis on pathological examination in November 2020. Patient continues to be asymptomatic at the present date with no disease progression seen on repeat CT scan and chromogranin serum level analysis.

This work adheres to the SCARE criteria [7]. A 51-year-old man with hypertension and prior aortic valve replacement presented to the emergency department with a 2-day history of right upper quadrant abdominal pain, nausea, and vomiting. Physical examination revealed a positive Murphy’s sign without jaundice or other specific findings suggestive of gallbladder malignancy. Laboratory tests showed elevated C-reactive protein and leukocytes. Based on a clinical diagnosis of acute cholecystitis, he underwent laparoscopic cholecystectomy. Intraoperative findings were unremarkable, with no excessive bleeding or liver complications. The postoperative course was uneventful, and the patient recovered in a standard ward without intensive care. The gallbladder specimen underwent routine histological evaluation. Grossly, a 7-mm greyish-white nodular thickening was noted at the cystic duct margin, with no other abnormalities. Microscopic examination of this nodule revealed a neuro-endocrine-like tumor made of round-to-oval cells organized in cords, nests, trabeculae, and rare tubules within a fibrous stroma and vascular network (Fig. 1a). Cells were monotonous, with uniform nuclei, inconspicuous nucleoli, and stippled chromatin (Fig. 1b). The mitotic index was low (1/10 HPF), with no necrosis or vascular/perineural invasion. The tumor invaded the subserosa (pT2). Immunohistochemistry showed positivity for chromogranin A, synaptophysin, and CD56 (Fig. 2a-b) with a Ki-67 index of 1 %, confirming a grade 1, pT2a NET with a positive cystic duct margin per WHO classification [5]. Systemic staging with thoracic and abdominal CT revealed no distant metastases but showed a few mildly enlarged celiac mesenteric and retroperitoneal lymph nodes, with no significant adenopathy in the pericholedochal region (Fig. 3). Gastric and colonic endoscopy were performed and were normal except for the presence of colonic diverticula, which was not accessible for biopsy due to its intramural location. A complementary resection of the cystic duct with regional lymphadenectomy was performed, showing no residual tumor. No adjuvant therapy was administered. The patient underwent follow-up for 5 years (from to date), with clinical assessments, abdominal CT scans (Fig. 3, Fig. 4), and chromogranin A evaluations conducted twice a year during the first two years and annually thereafter. No recurrence was observed at 5 years (Fig. 4).

## 3 Discussion

Neuroendocrine tumors arise from enterochromaffin cells, which are sparse in the gallbladder and biliary ducts, making NETs in these locations exceptionally rare (<0.5 % of all NETs) [2,3,5,6,8]. Their etiology is speculative, potentially linked to metaplasia from chronic inflammation, multipotent mesenchymal stem cells, ectopic pancreatic tissue or genetic syndromes [2,6,9,10]. Mixed tumors with adenocarcinoma components (MINEN) have also been described [2,5,6]. In this case, histopathology confirmed acute-on-chronic cholecystitis without metaplasia, adenocarcinoma or apparently predisposing lesion. NETs may occur sporadically or in association with syndromes like Von Hippel-Lindau or MEN1, with genetic alterations varying by subtype such as MEN1, DAXX, ATRX in well-differentiated NETs and TP53, RB1 in neuroendocrine carcinomas [NECs] [5,6,10].

Clinically, NETs are often asymptomatic or nonspecific, detected incidentally post-cholecystectomy, as in this described case [3,6,8,10]. In rare instances, large functional GB-NENs may cause symptoms such as diarrhea and flushing, which can facilitate preoperative diagnosis [8,11]. Imaging has also limited diagnostic value for GB-NEC [2,6,12]. Indeed, radiological features are nonspecific and no different from those of other gallbladder tumors [12]. Ultrasound typically reveals a solid, heterogeneous, hypoechoic mass. On non-contrast CT scans, these lesions often present as hypodense areas. Following contrast administration, CT imaging may show heterogeneous enhancement along with features such as cystic changes and necrosis [12]. Definitive diagnosis is mostly made during routine histopathological examination of cholecystectomy specimens, emphasizing histopathology’s diagnostic role [2,8,12].

Grossly, NETs present as small (<2 cm), greyish-white or yellowish submucosal nodules, as observed in our case at the cystic duct margin [5,6,8,[10], [11], [12]]. Microscopically, well-differentiated NETs exhibit nests, cords, and trabeculae of monotonous cells with “salt-and-pepper” chromatin, graded (G1–G3) by mitotic and Ki-67 indices [5,6,8,10,11]. The NET described in this case was assessed of low grade (G1) considering the low mitotic index (1/10 HPF) and the low Ki-67 (1 %) contrasting with the more aggressive behavior of neuroendocrine carcinomas (NECs) [9,13]. Immunohistochemistry, requiring positivity for at least two neuroendocrine markers (e.g., synaptophysin, chromogranin A), is the most effective tool for the diagnosis [5,6,8,13].

Differential diagnoses include extension from pancreatic or hepatic NETs, gangliocytic paraganglioma, or glomus tumors, none of which were supported in this case report [2,6].

Given the very low incidence of gallbladder NETs, the management of these tumors is not clearly codified, and requires their staging according to the Tumor Node Metastasis classification [2,3,6,[11], [12], [13]]. Radical surgery remains the only curative treatment option, with the choice of procedure guided by the established surgical guidelines for gallbladder cancer [1,2,6,8]. As in this described case, in patients with T2N0M0 tumors, basic cholecystectomy and gallbladder bed cautery may be sufficient [2].

Systemic chemotherapy is primarily indicated for NEC and metastatic forms of gallbladder NEN [8,12]. Prognosis is dependent on the tumor’s stage and grade, with well-differentiated NETs typically associated with a favorable outcome, while poorly differentiated NECs are more likely to present with metastases and carry a poorer prognosis [2,6,8,12,13]. As in this case report, most patients with G1-G2 NET have prolonged survival with no recurrence or metastasis [2,6,8]. However close monitoring is still necessary.

## 4 Conclusion

Gallbladder NETs are very rare, with uncertain etiology and variable prognosis. This incidental finding via routine histopathology underscores systematic specimen evaluation’s importance and the need for continued reporting of such cases to refine management protocols and prognosis.

## Author contribution

All the authors read and approved the final version of the manuscript.

Sarra Ben Rejeb (MD): conception, acquisition of data, literature research and preparing the manuscript.

Yasmine Chaabane (MD): conception, literature research supervision and revising the manuscript.

Moez Sahnoun (MD): Imaging data acquisition, revising and editing.

Adnen Chouchen (Pr): Supervision and revision.

## Registration of research studies

Not registered.

## Consent

Written informed consent was obtained from the patient for publication of this case report and accompanying images. A copy of the written consent is available for review by the Editor-in-Chief of this journal on request.

## Consent for publication

Written informed consent for publication of patient’s clinical details and clinical images were obtained from the patient.

## Ethical approval

The ethics approval is not required for case reports deemed not to constitute research at my institution “Hospital of FSI Tunis, Tunisia”.

## Guarantor

Dr. Sarra Ben Rejeb.

## Funding

The authors declare that there is no funding.

## Conflict of interest statement

The authors report no conflict of interest.
